# Fully understanding the efficacy profile of the COVID-19 vaccination and its associated factors in multiple real-world settings

**DOI:** 10.3389/fimmu.2022.947602

**Published:** 2022-10-25

**Authors:** Yunes Panahi, Behzad Einollahi, Fatemeh Beiraghdar, Mohammad Darvishi, Saeid Fathi, Mohammad Javanbakht, Sepehr Shafiee, Reza Akhavan-Sigari

**Affiliations:** ^1^ Pharmacotherapy Department, Faculty of Pharmacy, Baqiyatallah University of Medical Sciences, Tehran, Iran; ^2^ Nephrology and Urology Research Center, Baqiyatallah University of Medical Sciences, Tehran, Iran; ^3^ Infectious Diseases and Tropical Medicine Research Center (IDTMRC), Department of Aerospace and Subaquatic Medicine, AJA University of Medical Sciences, Tehran, Iran; ^4^ Department of Parasite Vaccine Research and Production, Razi Vaccine and Serum Research Institute, Agriculture Research, Education and Extension Organization (AREEO), Karaj, Iran; ^5^ Department of Medicine, Shahid Beheshti University of Medical Sciences, Tehran, Iran; ^6^ Department of Neurosurgery, University Medical Center Tuebingen, Tuebingen, Germany; ^7^ Department of Health Care Management and Clinical Research, Collegium Humanum Warsaw Management University, Warsaw, Poland

**Keywords:** COVID-19, vaccine, immunity, effectiveness, efficacy

## Abstract

We performed a review study according to recent COVID-19 vaccines’ real-world data to provide comparisons between COVID-19 vaccines regarding their relative efficacy. Although most vaccine platforms showed comparable effectiveness and efficacy, we highlight critical points and recent developments generated in studies that might affect vaccine efficacy including population-dependent effects of the vaccine (transplantation, adiposity, and specific comorbidities, as well as older age, male sex, ethnicity, and prior infection), vaccine type, variants of concern (VOC), and an extended vaccine schedule. Owing to these factors, community-based trials can be of great importance in determining vaccine effectiveness in a systematic manner; thus, uncertainty remains regarding vaccine efficacy. Long immune protection of vaccination with BNT162b2 or ChAdOx1 nCoV-19 has been demonstrated to be up to 61 months and 5–12 months after the previous infection, and boosting infection-acquired immunity for both the first and second doses of the BNT162b2 and ChAdOx1 nCoV-19 vaccines was correlated with high and durable protection. However, large cohort and longitudinal studies are required for the evaluation of immunity dynamics and longevity in unvaccinated, vaccinated, and infected individuals, as well as vaccinated convalescent individuals in real-world settings. Regarding the likelihood of vaccine escape variants evolving, an ongoing examination of the protection conferred against an evolving virus (new variant) by an extended schedule can be crucial.

## Introduction

Since the global pandemic of COVID-19, several COVID-19 vaccines have been developed and granted emergency use licenses to cover susceptible populations. According to World Health Organization (WHO), as of December 2021, nine vaccines have been approved for emergency use, utilizing diverse platforms, namely, Moderna (mRNA-1273), BNT162b2 (Pfizer/BioNTech), Ad26.COV2.S (Johnson & Johnson), AZD1222 (Oxford/AstraZeneca), COVISHIELD (Serum Institute of India; Oxford/AstraZeneca formulation), NVX-CoV2373, Novavax, BBIBP-CorV (Sinopharm, Beijing), and CoronaVac (Sinovac) (1). Nonetheless, achieving global vaccine coverage is a major barrier, i.e., the scale and rate of the vaccine rollout.

Phase III clinical trials reported high vaccine efficacy for RNA vaccines (BNT162b2 and mRNA-1273), followed by viral vector vaccines (AZD1222; Oxford-AstraZeneca) and inactivated virus vaccines (CoronaVac) (2–5). Following the test results of trials, precise and comparative questions have now been raised by the public, policymakers, and researchers (e.g., adverse events, dosing, and boost intervals, as well as quality, quantity, and durability of immune responses) as a result of the real and extraordinary challenges of mass vaccination rollout over the course of a period (6).

Real-world vaccine effectiveness has been reported for mainstream vaccines on the market by a series of studies in real-world settings (7, 8) as results of trials may be affected by different settings, e.g., the difference in the general population (younger, healthy adults in comparison with at risk of severe disease). There are various advantages and disadvantages in terms of safety profile, efficacy, immunogenicity, and immunity durability in the studies, where head-to-head trials for vaccine effectiveness (head-to-head comparisons) are of great importance to adjust different factors such as individual associated factors. Response to vaccination varies by age and timing depending on the type of vaccine and previous infection (9). As vaccination campaigns continue to expand, a growing body of evidence in real-world settings shows the effectiveness of vaccination in reducing transmission, severe disease, and death. Such a dataset as input allows the epidemiologist to examine raised questions (10, 11). Evidence of positive vaccination along with more effective public health communication strategies can be capable of boosting public trust and countering COVID-19 vaccine misinformation (11, 12).

**Figure 1:**
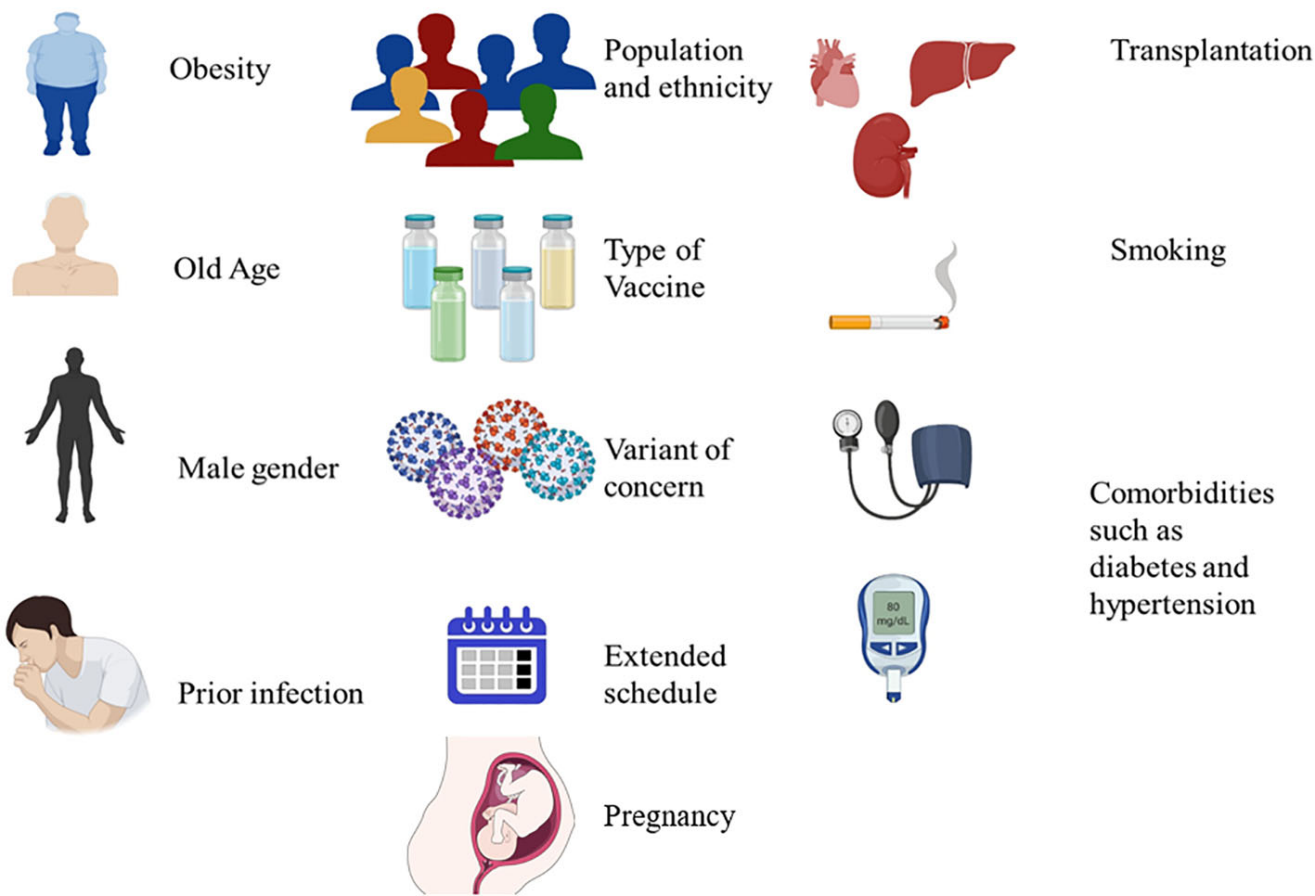
Factors affecting the effectiveness of the vaccine.

The present study aimed to cover recent COVID-19 vaccines’ real-world data on vaccine effectiveness and efficacy as follows: we addressed the role of individual factors (transplantation, adiposity, smoking, and specific comorbidities, as well as older age, male sex, ethnicity, and prior infection), vaccine type, variants of concern (VOC), an extended vaccine schedule, and longevity of protection (the heterogeneity and longevity of neutralizing antibody) in higher boosting and better protection in real-world settings to provide vaccination figures for prioritizing the administration mass vaccination rollouts in a way that minimizes morbidity and mortality of COVID-19 disease (**Figure 1**); the article outlines the significance and limitations of investigations for determining vaccine effectiveness and important groups for whom additional doses of the vaccine may be beneficial.

## Vaccine and study types

Most studies have been conducted to evaluate the real-world effectiveness of COVID-19 vaccines, especially mRNA vaccines, in high-income countries using data from reliable and interlinked databases, whereas such databases are often not available in low- and middle-income countries (LMICs), resulting in the lack of such studies in these countries. Studies such as cohort studies, test-negative design case-control studies, and observational studies have identified major limitations, including short follow-up, limited evaluation, and mitigation of potential confounders (e.g., previous SARS-CoV-2 infection and healthcare-seeking behavior, sex, age, ethnicity/religion, geographical location, chronic disease and/or comorbidities, time, and socioeconomic status). In LMICs, given attention ought to be paid to prospective studies and management of confounders and missing data in resource-constrained settings (13).

As reported by a mathematical model calibrated to King County, WA, a certain COVID-19 vaccine may have efficacy on susceptibility (reduce susceptibility to SARS-CoV-2 infection upon exposure) (14), or COVID-19 disease-modifying vaccines may be capable of preventing COVID-19 disease by decreasing the likelihood of symptoms, which in turn result in asymptomatic infection with viral shedding, leading to ongoing transmission (15).

Observational cohort analysis in an eight-hospital system in Michigan reported that fully COVID-19 vaccinated patients rarely experience emergency visits and hospitalizations as compared with unvaccinated individuals, even in areas with a high incidence of variants. Elderly immunized patients are hospitalized with comorbidities. In populations with comorbidities, the risk of developing severe outcomes has been reported to be higher regardless of vaccination. Evaluating the effectiveness of vaccination based on the type of vaccine will give a deeper understanding of the effectiveness of vaccines as many mutations are evolving (16).

**Figure 2:**
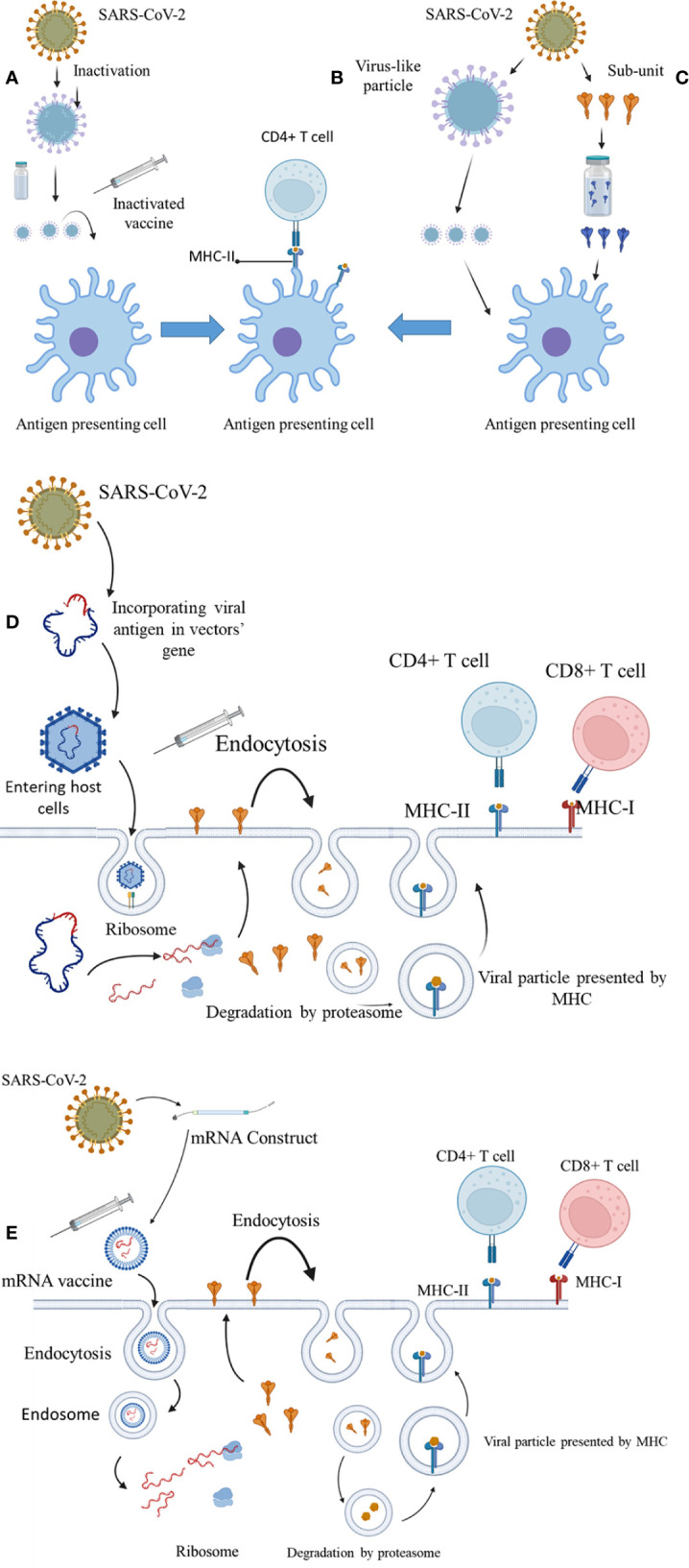
The immunogenicity mechanisms of COVID-19 vaccines. **(A)** Inactivated virus. **(B)** Virus-like particles. **(C)** Spike protein subunit. **(D)** Vector-based vaccines. **(E)** mRNA construct–based vaccines.

Currently, different vaccines are available with different mechanisms for immunogenicity (**Figure 2**). These vaccines prevent contraction or adverse outcomes of COVID-19 by increasing the production of naturalizing antibodies, production of cytokines, and activation of cytotoxic t-cells. mRNA vaccines activate the IFN-γ CD4^+^ and CD8^+^ T cells and upregulate the production of IL-2 (17). It has been suggested that their immunogenicity is mainly due to responses to the activation of T_H_1 cells. The activation of CD4^+^ cells, CD8^+^ T cells and cytokines such as TNF and IFN-γ also play a crucial role in the immunogenicity of viral vector vaccines (18). On the other hand, the main immune system response of subunit-based vaccines is through the activation of CD4^+^ cells (19). However, it has been shown that immune system responses to different vaccines with similar immunogenicity mechanisms can slightly differ from other vaccines. As a result, it is necessary to evaluate the efficiency of the vaccines based on real-world data (19).

Based on real-world evidence, COVID-19 vaccines are thought to be more effective than expected (2–4, 20–25). Synthesized evidence demonstrated 91.2%, 98.1%, and 65.7% effectiveness for the BNT162b2 (Pfizer-BioNTech), mRNA-1273 (Moderna), and CoronaVac vaccines, respectively (26).

Effectiveness of 96.2% and 98.2% has been determined for the BNT162b2 (Pfizer-BioNTech) and mRNA-1273 (Moderna) based on real-world analyses (21). The high (>50%) or very high (>80%) effectiveness of two doses of the BNT162b2, AZD1222 (AstraZeneca), mRNA-1273, HB02 (Sinopharm), and Gam-COVID-Vac (Sputnik V) vaccines have been revealed among Hungarian individuals, where their ability to prevent SARS-CoV-2 infection (estimated adjusted effectiveness: 68.7%–88.7%) and COVID-19–related death (87.8%–97.5%; more than 9,500 deaths) was reported to be ≥7 days after the second dose (27).

Based on a study in Chile, adjusted effectiveness rates of Sinovac SARS-CoV-2 vaccine in preventing SARS-CoV-2 infection and COVID-19–associated deaths have been reported to be 65.9% and 86.3% ≥14 days after the second dose, respectively, among more than 10 million people (24). The high effectiveness of the mRNA-1273 has been determined according to clinical trials and real-world settings (28, 29).

Different effectiveness of vaccines in real-world settings may be associated with patients’ differences (differences in comorbidities, outcomes definitions, age, etc.) (30).

Despite the unbiased estimates of efficacy in clinical trials, there is a possibility that trial participants may differ from the general population, especially regarding the differences between real-world deployment of COVID-19 vaccines and authorized dosing schedules. Community-based trials (large community-based studies) can be of great importance in determining vaccine effectiveness (31) when performed independently of the vaccination status and symptoms in a systematic manner. A large community-based survey in the United Kingdom demonstrated that the BNT162b2 and ChAdOx1 vaccines had favorable effectiveness after two doses and against symptomatic and high viral load.

Sub-populations (32, 33) or symptomatic testing programs (10) could result in bias from vaccination status that affects the test-seeking behavior of those who do not require healthcare (34).

BNT162b2 was found to be capable of inducing a robust immune response, as real-world data confirmed vaccine effectiveness after full immunization (10, 23, 35, 36).

The comparative protection of two doses of BNT162b2 and ChAdOx1 against COVID-19 endured over 6 months in the fully vaccinated individuals during the Delta variant peak, indicating that there is no differential waning of immunity between BNT162b2 and ChAdOx1. Continuous monitoring of the various vaccines’ effectiveness against variants is needed if SARS-CoV-2 variants impose any impact on SARS-CoV-2 vaccine effectiveness for booster and vaccine combinations strategies (37).

Lower protection and antibody titer of ChAdOx1 vaccine have also been documented compared with BNT162b2 (38–43), which can be linked to a shorter interval to a decline in titer, when long-interval regimens have been found to be associated with higher antibody, B-cell, and T-cell responses, as well as higher vaccine effectiveness against symptomatic COVID-19 (40, 44, 45), suggesting a higher possible threshold to prevent all SARS-CoV-2 compared with symptomatic COVID-19 (46). Thresholds for protective antibody titers are needed to correctly estimate vaccine*-*induced immune response to prevent re-infection (46).

Biased estimates of vaccine comparative effect may be occurred due to inadequate control for vaccination dates where variation in community transmission is seen over time. Observational data may be prone to confounding by indication and differential testing rates between vaccinated and unvaccinated individuals, suggesting the minimization of the effect of such differences (37, 47).

It is noteworthy that caution should be taken in interpreting the superiority of one vaccine over another based on evidence of antibody responses alone (48) when adjustment of different factors such as individual associated factors is needed through head-to-head trials for vaccine effectiveness (head-to-head comparisons). Response to vaccination varies by age and timing depending on the type of vaccine and previous infection (9).

It is noteworthy that Moderna and BNT162b2 vaccines’ effectiveness [single dose: 61.0% (95% CI, 31.0%–79.0%); after the second dose: 80.0% (95% CI, 91.0%–56.0%)] against infectiousness to others has been reported by randomized controlled trials and a retrospective cohort study, respectively (49, 50), but the determination of reliable effectiveness for every vaccine is required based on the vaccination coverage as the control such as the procedure applied in the SARS-CoV-2 Immunity and Reinfection Evaluation (SIREN) study (51).

## Extended schedule

Partial vaccination is less effective against COVID-19 disease, which is associated with hospitalization, ICU admission, and death when compared with full vaccination. Higher effectiveness of BNT162b2 and AZD1222 (AstraZeneca) against SARS-CoV-2 infection has been achieved by applying an extended schedule of vaccination in England, resulting in increased boosting and better protection against variants in comparison with short-interval schedules (45). In addition, higher vaccine effectiveness has been reported for longer periods after vaccination, whether for partial or complete vaccinations (52, 53). An extended schedule of vaccination (e.g., extending the dosing interval) is warranted to be further evaluated for optimizing vaccine coverage and determining protection against SARS-CoV-2 new variants (7, 54). It should be taken into consideration that schedules and handling/administration of vaccines (type of vaccine, doses and timing between them, and heterologous prime-boost) can be involved in reducing or decreasing vaccine effectiveness (55).

## Individual factors, variants of concern (VOC), and variants of interest (VOI)​​

Data from 212,102 vaccinated individuals with at least one dose of BNT162b2 and ChAdOx1 in England showed that antibody positivity (positive IgG findings on the LFIA, presence, and declining levels of neutralizing antibody titers) was at lower levels in individuals with transplantation, adiposity, smoking, and specific comorbidities, as well as older age and male sex, indicating the need of the second booster dose. Age, sex, prior infection, obesity, comorbidities, and vaccine type have been described to be of great importance in antibody response, especially after the first doses. Both vaccines revealed a marked increase in subjects with detectable antibodies and little evidence of a subsequent decrease after the second dose (48).

Many countries prioritized high-risk groups (elderly and healthcare workers) for vaccination (43, 56). A 6-month longitudinal prospective study has documented a decreased level of humoral response 6 months after receiving the second dose of the BNT162b2, particularly among men, older age individuals (65 years or older), and immunosuppressed healthcare workers (57).

Lower antibody positivity has been reported 3–4 weeks after the second dose of ChAdOx1 in individuals aged over 70 years based on the REACT-2 program in England. Antibody positivity of the BNT162b2 vaccine has been reported to be >90% in all age groups except 75 years and older (48) as several studies reported age-dependent immune response, when lower frequencies of neutralizing antibodies have been widely documented following BNT162b2 vaccination in the elderly population against SARS-CoV-2 (58), as well as B.1.1.7 (Alpha), B.1.351 (Beta), and P.1. (Gamma) VOC (59, 60). The main mutant SARS-CoV-2 variants are described as B.1.1.7, B.1.351, P.1, B.1.617.2 (Delta), and B.1.1.529 (Omicron)

Lower levels of neutralizing antibodies were found in the elderly 22 days after the first dose of BNT162b1 (61). After the first dose, no neutralizing activity was observed for P.1 and B.1.1.7, and a sharp decrease was reported at the age of 80 years (59), but concomitantly diminished protection needed investigation. Synthesized real-world evidence demonstrated lower efficacy of the vaccine in the elderly because of immunosenescence and comorbidities, whereas protective effect has been observed in the healthcare workers (26). Lower frequencies of neutralizing antibodies have been attributed to quantity (lower concentrations of antibodies) and quality (lower-affinity antibodies) linked to B-cell selection, decreased CD4^+^ T cells, or intermingling of both (59). On the other hand, VOC has been associated with a majority of breakthrough infections (60). Thus, considering the combined effects of VOC and lower frequencies of neutralizing antibodies is of particular importance for decision-making about booster vaccinations, especially in older aged persons as a high-risk population (59). Vaccine effectiveness estimates against SARS-CoV-2 variants need future studies (62).

Declining antibody positivity prior to second doses has been described to be linked to infection vulnerability (63). Decreased levels of neutralizing antibodies increase the likelihood of symptomatic infection (64). This issue becomes even more pronounced when the Delta variant can somewhat escape monoclonal antibodies and neutralizing polyclonal antibodies caused by prior SARS-CoV-2 infection or vaccination with a single dose of the BNT162b2 or the AZD1222 vaccine, and neutralization of this variant has been occurred in about 10% of the sera, suggesting the second dose of vaccination for higher protective titer. In this regard, a two-dose vaccination could induce remarkable levels of neutralizing antibody titer against the B.1.1.7, B.1.351, and B.1.617.2 variants 8 to 16 weeks after vaccination (63).

A study showed increased protection from symptomatic COVID-19 disease in individuals aged over 50 years using a booster dose of BNT162b2, regardless of which initial course of BNT162b2 or ChAdOx1-S was used in the United Kingdom (65).

Effectiveness after one dose of BNT162b2 or AZD1222 has been also estimated to be remarkably lower in individuals with the B.1.617.2 variant (30.7%; 95% CI, 25.2–35.7) when compared to those with the B.1.1.7 variant (48.7%; 95% CI, 45.5–51.7). This difference has been attributed to 11.9% for the BNT162b2 vaccine and 18.7% for the AZD1222 vaccine, whereas vaccine efficacy against both the B.1.617.2 and B.1.1.7 variants increased after the second dose of BNT162b2 (B.1.1.7: 93.7%; B.1.617.2: 88.0%) and ChAdOx1 by 74.5% (B.1.1.7: 67.0%; B.1.617.2: 88.0%), indicating the importance of vaccine uptake maximization (43).

It has been reported that two doses of the CoronaVac vaccine had 59.0% effectiveness against the B.1.617.2 variant (95% CI, 16.0–81.6%) (66). Regarding prevention of moderate to severe COVID-19, vaccine effectiveness for single doses of Ad26.COV2.S was 66.2% and 68.1% at least 14 and 28 days after vaccination against the P.1 variant, respectively (67). The Ad26.COV2.S vaccine efficacy against severe to critical COVID-19 was 81.9% and 87.6% at 14 and 28 days after vaccination, respectively (67). Regarding B.1.351, vaccine efficacy of Ad26.COV2.S was 52.0% and 73.1% at least 14 and 28 days after vaccination against moderate to severe to critical illness (B.1.351), the vaccine efficacy was 64.0% and 81.7% at least 14 and 28 days after vaccination, respectively, among severe to critically ill patients (67).

A study indicated that vaccine efficacy of ZF2001, an RBD subunit vaccine, was 92.7% and 88.3% against the Alpha variant in the short-term and long-term follow-up, respectively; efficacy against the Delta variant was 81.4% and 76.1%, respectively, and efficacy against the Kappa variant of interest was reported to be 84.8% and 75.2%, respectively. This study demonstrated the effectiveness of ZF2001 in the prevention of symptomatic and severe to critical diseases for 6 months after full vaccination (68).

Boosted vaccinees with mRNA vaccines showed potent neutralization of B.1.1.529 mutant, only geometric mean neutralization titer (GMNT) of 4–6-fold lower than wild type (6-fold for mRNA-1273 and 4-fold for BNT162b), indicating increased cross-reactivity of neutralizing antibody, while this decrease was 17-fold for Ad26.COV2.S. In addition, among recently vaccinated people (<3 months), GMNT decreased by 43-fold for mRNA-1273 and by 122-fold for BNT162b (69).

mRNA-1273 has demonstrated more efficiency (higher neutralizing antibody levels) in the prevention of COVID-19 when compared with BNT162b2 or ChAdOx1 (70–72). However, a third booster dose of BNT162b2 or mRNA-1273 may be associated with increased antibody levels (4-fold) against the ancestral D614G variant and higher neutralizing antibodies against the Omicron BA.1 variant and the Delta variant, supporting proper protection against the Omicron variants (70).

Heterologous BnT162b2 vaccine booster was exhibited to be capable of producing higher neutralizing titers against the SARS-CoV-2 Omicron BA.2 variant. Further studies are needed to assess the neutralization activity of vaccine regimens against the Omicron sublineages, e.g., other mRNA vaccines, adenovirus-vectored vaccines, recombinant vaccines, and inactivated vaccines (73).

Regular inactivated vaccines are capable of inducing a protective effect on the severity of clinical presentation of Omicron BA.2 infection through antibody response, but waning protection has been revealed over time (74). In addition, regular and booster immunization with inactivated vaccines could be capable of increasing neutralizing abilities against the Omicron variant both in breakthrough infections and vaccines (75), suggesting the potential effectiveness of booster vaccination of children in the future (74).

Vaccinated individuals and subjects with prior infection demonstrated less neutralizing activity for both the B.1.617.1 and B.1.617.2 variants when compared with WA1/2020; however, detectable neutralizing activity was found to be above the threshold of detection for both the B.1.617.1 and B.1.617.2 variants 3 months after infection or after the second dose of COVID-19 mRNA vaccines, indicating retainment of protective immunity against both variants (76). The Omicron type BA.1 has been reported to have potent evasion properties (evasion of neutralizing antibody responses) (77–79), suggestive of lack of neutralization against the Omicron variant (80), that reveals little cross-reactive responses for neutralizing antibodies with the earlier variants; thus, unvaccinated individuals suffering from the Omicron BA.1 variant (without prior infection) do not develop adequate protection against SARS-CoV-2 variants other than Omicron BA.1, indicating the need of vaccination for full protection (77).

Ward et al. have confirmed higher antibody positivity of both BNT162b2 and ChAdOx1vaccines among female participants (48). Women and young age have been attributed to a higher capacity to generate humoral immune responses for the BNT162b2 vaccine (81), whereas a lower humoral response has been observed 6 months after receiving the second dose of the BNT162b2 vaccine among men (57).

Despite improvement in anti-spike antibodies after two doses of the SARS-CoV-2 mRNA vaccine series, transplant recipients are high-risk populations for COVID-19 (82). In accordance with the mentioned study, data from a cohort of healthcare workers demonstrated lower neutralizing antibody titers in immunosuppressed healthcare workers during the peak and end-of-study periods (54), which was similar to other results presented previously (83, 84).

Obesity, hypertension, smoking, and specific comorbidities have been reported to be linked to lower antibody response after vaccination with BNT162b2 and ChAdOx1 (43, 85). However, comorbidities have been described to be influenced by shielding behavior (48).

A less virulent and highly transmissible variant may be associated with a higher risk for older individuals and those with comorbidities (e.g., immunosuppression) or unvaccinated individuals (86).

Conflicting results reported higher neutralizing antibody titer in obese persons during long-term follow-up (57, 87). Vaccinated obese individuals should be evaluated for determining a higher or lower risk of the breakthrough, and the protectivity of the vaccine among them, if there is a high humoral response. Otherwise, lower antibody response raises concern in these populations suffering from COVID-19 regarding their poor outcomes. Underlying conditions such as diabetes as well as liver and chronic kidney diseases were also linked to lower antibody response after vaccination (43). Overwhelming evidence indicates that at-risk groups should be prioritized to boost vaccine effectiveness.

Post-vaccine seropositivity was found to be associated with Black and Asian ethnicity 14–60 days after the first ChAdOx1 or BNT162b2 vaccination in the United Kingdom, where 20,505 (82.1%) of 28,144 participants were seropositive post-vaccination (39). Higher antibody positivity was observed in Black and Asian ethnicity compared with the white race following the first-dose vaccination with ChAdOx1 or BNT162b2 (43). Black race (aRR 1.7, 95% CI, 1.3–2.2, p < 0.001) was significantly linked to seropositivity after vaccination with a two-dose regimen of BNT162b2 and ChAdOx1 or the Moderna vaccine in Irish hospital healthcare workers (88).

SARS-CoV-2 infection is linked to devastating effects in pregnancy including a higher rate of hospitalization and ICU admission, maternal death, preterm birth, stillbirth, etc. (89). On the other hand, the safety and effectiveness of COVID-19 vaccines during pregnancy are an important concern due to limited, conflicting, and changing advice on vaccine safety, resulting in vaccine hesitancy among this vulnerable population (90–92). Observational studies and large case series reported no adverse pregnancy effects of mRNA COVID-19 vaccination on pregnancy or neonatal outcomes (93–96). A systematic review and meta-analysis study demonstrated the effectiveness of COVID-19 vaccination in pregnancy, without increasing the risk of adverse effects in 23 studies containing 117,552 COVID-19–vaccinated pregnant individuals with mRNA vaccines (97).

To determine whether COVID-19 vaccination should be performed for pregnant women, high-quality robust data are required, pending updates from reliable and interlinked databases (national database) and the findings of ongoing trials with a prospective approach, and active post-release monitoring for any rare adverse effects.

## Prior infection and longevity of protection

Waning immunity has been revealed over time in people receiving COVID-19 vaccines (98, 99). Regarding the longevity of protection, two doses of the BNT162b2 vaccine were able to decrease the risk of asymptomatic and symptomatic infection from 14 to 73 days after the second dose (adjusted effectiveness: 89%; 95% CI, 78–94), but the reduction of adjusted vaccine effectiveness was observed at a median of 238 days (53%; 95% CI, 28–69) during a period of Delta variant predominance, indicating the importance of booster doses. On the other hand, the adjusted effectiveness of AZD1222 was found to be reduced (58%; 95% CI, 23–77) 14–73 days after receiving the second dose as compared to BNT162b2, but no considerable difference was found after long periods of the use of the second dose (51).

Based on the findings of a study in Qatar, mRNA vaccines BNT162b2 and mRNA-1273 exhibited moderate and short-lived protection against symptomatic BA.1 and BA.2 Omicron, but protection declined to low levels 4 months after the second booster dose. Rebounded effectiveness was revealed in the first month after the booster vaccination. Overall, mRNA vaccines showed durable and robust protection against hospitalization and death after the second dose (100), supporting such durability of protection for at least several months after the second booster dose (100, 101), and the importance of booster vaccination.

By using a nationwide linked database of Brazil, it has been demonstrated that heterologous CoronaVac plus a BNT162b2 booster vaccination contributed to robust and durable protection against hospitalization or death of Omicron for at least 120 days [vaccine effectiveness: 84.1% (95% CI, 83.2–84.9)] except for subjects aged over 80 years (102)

On the other hand, given the correlation of prior SARS-CoV-2 infection with higher antibody responses (39, 43, 103), boosting of infection-acquired immunity by both the first and second doses of BNT162b2 or AZD1222 has been demonstrated to be associated with high and durable protection (combined protection of >90%) after 1 year of primary infection (51), that is, in line with other findings reported higher protection in vaccinated individuals with prior infection (104). Whereas infection-acquired immunity waned in unvaccinated persons after this period. In addition, controversial findings showed the superiority of infection-acquired immunity and vaccine-acquired immunity (105–109), as well as their equivalency (110). Finally, a growing body of evidence suggests the potential for recommending boosting infection-acquired immunity by vaccination for previously infected individuals (46, 111).

Long immune protection has been reported to be up to 61 months and 5–12 months after the previous infection (9, 112–114), which is in need of further evaluation of change and duration of immunity in unvaccinated, vaccinated, and infected individuals as well as convalescent individuals who got vaccinated. Long-term protection against COVID-19 by immunization remains to be addressed, where an in-depth understanding of the proper long-term antibody dynamics is limited by the restricted patient groups, the short follow-up time, or the availability of accurate virus-based detection of the neutralizing antibody (115).

The association of lower disease severity with the presence of SARS-CoV-2–specific CD4^+^ and CD8^+^ T cells has been revealed (116, 117). Regarding elicitation of T-cell responses by COVID-19 vaccines (118–120), in addition to neutralizing antibodies (121–123), two doses of AZD1222 vaccine were found to be capable of eliciting antiviral polyfunctional spike protein–specific T_H_1 (CD4^+^ and CD8^+^ T cell) with a diverse T-cell receptor (TCR) repertoire in all adult age groups, which may be linked to long-lasting protection against SARS-CoV-2 variants responsible for severe diseases (123). Memory T cells elicited by BNT162b2 have been demonstrated to respond to the Omicron variant with preservation of polyfunctionality, suggesting effector functions of these cells in vaccinated peoples (124).

The time-dependent waning of antibodies or T cells in individuals after infection or vaccine administration is of particular importance. Thus, the effect of novel SARS-CoV-2 variants on humoral immunity should be evaluated (several studies reported); in addition, T-cell responses to the new variants need further monitoring.

Neutralizing antibodies in COVID-19 convalescents have been shown to persist for up to 1 year after the onset of symptoms, with reduced neutralizing antibody titers over time (125–127). Similar reduction rates for both binding and neutralizing antibody responses have been reported for mRNA vaccine during the first 7 months after vaccination (128, 129), as well as for infection.

A previous study demonstrated neutralizing responses up to 480 days (16 months) in convalescents of symptomatic COVID-19. Convalescents with asymptomatic infections showed a considerable rate of undetectable neutralizing antibodies (115). The dynamics and longevity of neutralizing antibody titers in convalescents can be affected by re-exposure to the virus (115).

However, the dynamics of the neutralizing antibody response in patients recovering from COVID-19 have shown varying levels if similar rates of waning are observed after vaccination or new VOC appear to reduce the effectiveness of the vaccine and immune longevity is only determinable at the individual level (130). Overall, the clinical concerns are the necessity of COVID-19 vaccination including the timing of vaccination in convalescents, the need for revaccination, and its timing for former vaccines.

## Conclusion

The present review aimed to cover the recent COVID-19 vaccines’ real-world data on vaccine effectiveness. Although most vaccine platforms showed comparable effectiveness and efficacy, herein, we provided strong evidence for the role of population-dependent effects of the vaccine (transplantation, adiposity, and specific comorbidities, as well as older age, male sex, ethnicity, and prior infection), vaccine type, VOC, and an extended vaccine schedule in vaccine effectiveness. It is worth noting that vaccination schedules and handling/administration of vaccines can be influential factors in reducing or increasing vaccine effectiveness.

Long immune protection of vaccination with BNT162b2 and ChAdOx1 nCoV-19 has been demonstrated to be up to 61 months and 5–12 months after the previous infection, respectively; however, evaluation of change and duration of immunity in unvaccinated, vaccinated, and infected individuals, as well as vaccinated convalescent individuals in real-world settings, can be capable of providing vaccination figures by increasing our understanding of accurate long-term antibody dynamics, suggesting the importance of large cohort and longitudinal studies for the evaluation of immunity dynamics and longevity.

Regarding the correlation of prior SARS-CoV-2 infection with higher antibody responses, a growing body of evidence suggests that boosting infection-acquired immunity for both the first and second doses of the BNT162b2 and AZD1222 vaccines was linked with high and durable protection. Regarding the likelihood of vaccine escape variants evolving, an ongoing examination of the protection conferred against an evolving virus (new variant) by an extended schedule can be crucial. The real-world effectiveness studies not only are capable of monitoring the impact of the vaccine but also are able to provide country-specific inputs for modeling, planning, budgeting effective vaccination strategies, and facilitating preventative action plans (non-pharmaceutical interventions) and control measures, especially for high-risk populations.

## Author contributions

YP, BE, FB, SF, SS, and MJ participated in the design of the review, made a discussion, edited the manuscript, wrote the paper, and contributed to figures. MD and RA-S designed the revision, revised the draft, and edited the paper. All authors read and approved the final manuscript.

## Conflict of interest

The authors declare that the research was conducted in the absence of any commercial or financial relationships that could be construed as a potential conflict of interest.

## Publisher’s note

All claims expressed in this article are solely those of the authors and do not necessarily represent those of their affiliated organizations, or those of the publisher, the editors and the reviewers. Any product that may be evaluated in this article, or claim that may be made by its manufacturer, is not guaranteed or endorsed by the publisher.
